# Supplementary material for: Protease and DNase Activities of a Very Stable High-Molecular-Mass Multiprotein Complex from Sea Cucumber Eupentacta fraudatrix
Source: Int J Mol Sci. 2022 Jun 15;23(12):6677. doi: 10.3390/ijms23126677 (PMC9224385; doi:10.3390/ijms23126677)
Supplement: Supplementary file 1 [file ijms-23-06677-s001.zip › ijms-1755835-supplementary.pdf]

Supplementary data

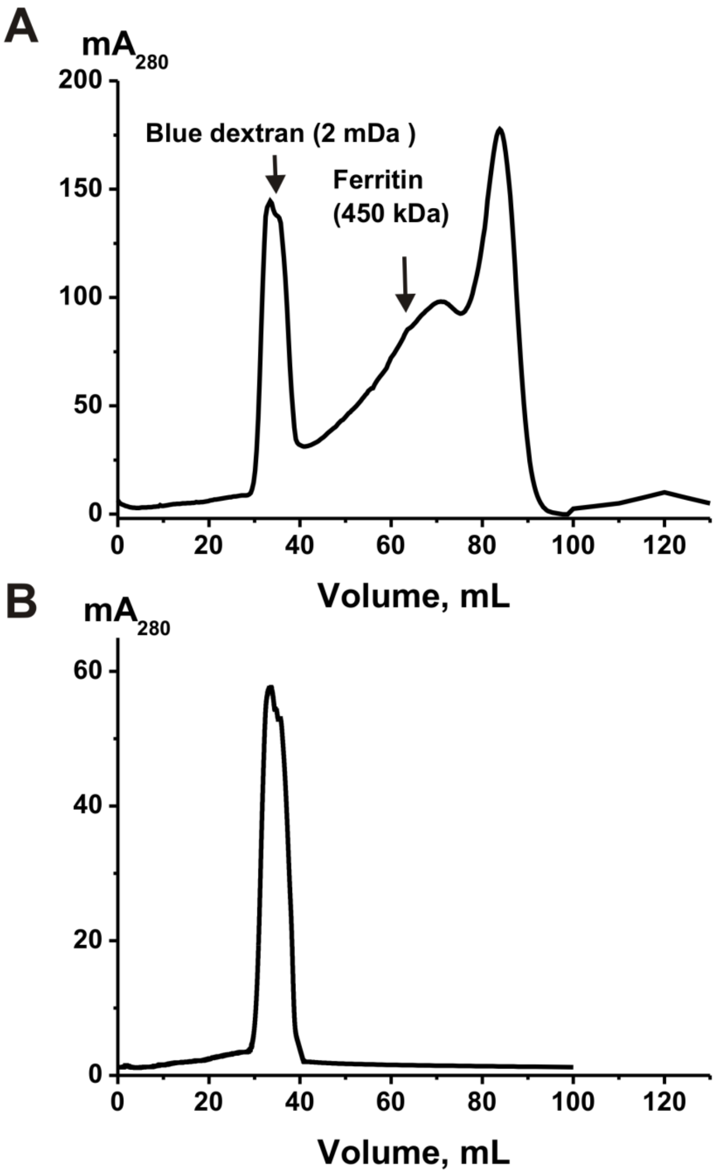

Supplementary Figure S1.

Isolation and of sea cucumber complex. Gel filtration of sea cucumber homogenate proteins on a Sepharose 4B column (A). Re-gel filtration of the purified complex (A) on the Sepharose 4B, ~2000 kDa (B). In all Panels, (—) - absorbance at 280 nm (A_{280}).
